# Are Patients Aware of Their Rights? A Cross-Sectional Study of Visitors to Three Primary Healthcare Centers in Riyadh, Saudi Arabia

**DOI:** 10.7759/cureus.19290

**Published:** 2021-11-05

**Authors:** Muath Al-Rebdi, Unaib Rabbani, Saeed M Alqahtani

**Affiliations:** 1 Department of Family Medicine, King Saud Bin Abdulaziz University for Health Sciences, Riyadh, SAU; 2 Family Medicine Academy, Qassim Health Cluster, Qassim, SAU; 3 Aviation Medicine Department, King Abdulaziz Medical City (KAMC) Ministry of National Guard Health Affairs, Riyadh, SAU

**Keywords:** ethical and legal principles in medical practice, saudi arabia, medical record confidentiality, patients’ bill of rights, patients’ rights

## Abstract

Background and objective

Patients’ rights are an essential aspect of human rights. Thus, in Saudi Arabia, the Ministry of National Guard Health Affairs (MNGHA) established a Patients’ Bill of Rights (PBR) and introduced it to the public about 30 years ago. This study aims to assess levels of awareness of PBR and to identify their information sources among visitors to primary healthcare centers (PHCCs).

Methods

This cross-sectional study included 358 visitors, aged 17 to 78, to three PHCCs at the National Guard Hospital in Riyadh, Saudi Arabia. The study was conducted between April and December 2017 using a self-administered questionnaire. The initial sample (convenience sampling) was refined to match the proportion of participants with the relative size of populations served by the centers. Descriptive statistics were used to determine the relationships between participants’ demographic data and levels of awareness of patients' rights.

Results

Most participants (72.2%) were moderately aware of their rights, but the majority (65.3%) were unaware of the existence of the PBR. Healthcare providers were the main information source for patients’ rights, although participants with the highest awareness scores obtained information from social media. Level of awareness was significantly associated with education, income, and regular hospital follow-ups.

Conclusions

The study results should motivate healthcare providers, stakeholders, and health organizations in Saudi Arabia to raise and maintain public awareness of patients’ rights and to implement them.

## Introduction

Health is a fundamental human right. The constitution of the World Health Organization (WHO) includes the principle that every human being should enjoy the highest attainable standard of health, regardless of race, religion, political belief, and socioeconomic status [1]. The United Nations adopted the Universal Declaration of Human Rights in 1948 [2]. One of the most essential human rights of the Declaration is access to healthcare without discrimination, and in 1977 the WHO presented a vision of healthcare for all to be achieved by 2000, prompting the establishment of patients’ rights internationally [3,4].

In response, ministries of health (MOHs) worldwide developed formal documents, including patients’ bills of rights (PBRs), to preserve the rights of patients and doctors. Saudi Arabia’s Ministry of National Guard Health Affairs (MNGHA) published a PBR in 1989 entitled Rights and Responsibilities of Patients and their Families [5]. Saudi Arabia’s MOH defined patients’ rights as “Policies and rules that must be preserved and protected by the health facility toward patients and their families”[6].

Although health organizations in most countries have published PBRs, studies conducted in the Middle East have reported poor awareness of PBRs among the general population, a lack of respect for patients’ rights, and dissatisfaction with the implementation of PBRs among healthcare providers. Kuzu et al [7], surveyed patients hospitalized in three large public hospitals in Turkey and found that only 9% knew about patients’ rights. In Iraq, Khalaf et al [8] reported that only 22.8% of patients knew of a national statement on patients’ rights. Yaghobian et al [9], concluded that 70% of Iranian patients knew little about the PBR’s content. However, few studies have assessed the awareness of patients’ rights and their application in Saudi Arabia. Almoajel [10], studied awareness of patients’ rights among hospitalized patients in Riyadh, and found that most knew nothing about Saudi Arabia’s PBR. Al-Mosa and Al-Ghamdi [11] reported that, in Jeddah, only 1.6% of patients attending chronic disease clinics at PHCCs had read the PBR. One of the first studies on this topic in Saudi Arabia was conducted by Albishiin [12]. He investigated awareness of patients’ rights among patients, nurses, and doctors, concluding that a lack of knowledge, inadequate implementation of rights at healthcare centers, and poor subsequent care all represented barriers to patients’ rights.

This study examines patients’ awareness of their rights and patients’ information sources, with the aim of improving healthcare systems, patient satisfaction, and patient-doctor relationships.

## Materials and methods

Study design and setting

This study was conducted at the National Guard Hospital in Riyadh, a modern hospital providing primary, secondary, and tertiary healthcare under the umbrella of the MNGHA. The MNGHA healthcare service ranks fourth among hospitals in the Arab world, according to the Ranking Web of Hospitals [13], providing primary healthcare to approximately 320,000 patients annually. This cross-sectional study was designed to investigate awareness of patients' rights among visitors to PHCCs. Using the software Open Epi Version 3, the minimum sample size needed to achieve a 95% confidence level was calculated to be 335 participants which was increased to 358 to compensate for incomplete questionnaire.

Primary healthcare centers and participants

Using convenience sampling, 358 individuals who attended three PHCCs at the National Guard Hospital were enrolled in this study. Sample was divided into three PHCC groups proportionate to the size of the population they serve:

· Health Care Specialty Center, about 200,000 (n=220 participants);

· King Abdul-Aziz Housing City Clinic, about 50,000 (n=58 participants); and

· Comprehensive Specialized Clinic, about 70,000 (n=80 participants).

Inclusion criteria and data collection

To be eligible for this study, individuals had to be Saudi national adults of either gender with the ability to give consent. Healthcare providers and hospital employees were excluded. The study (RC17/049/R) was approved by the ethics committee of the King Abdullah International Medical Research Center, Riyadh. The study was conducted between February and November 2017. Data were collected using a self-administered questionnaire, adapted from the MNGHA PRB and reviewed and approved by two expert consultants to ensure content validity. Participants provided verbal consent to participate after receiving an explanation about the nature and purpose of the study.

Study tool

The questionnaire comprised three sections. Patients’ demographic characteristics and awareness of the PBR are detailed in the Results section. The section also included 21 questions on participants’ awareness of their rights addressing the principal rights of the PBR, scored using a Likert scale (strongly agree, agree, neutral, disagree, or strongly disagree). The questions were scored 0-4 and enquired to what extent patients agreed or disagreed with a specific item of the PBR, with a total score of 0-84. Those with higher scores demonstrated better awareness of the PBR. Results were categorized into three levels: poor awareness (0-28), moderate awareness (29-56), and excellent awareness (57-84). The last section included two multiple-choice questions: to identify information sources for patients’ rights and assess participants’ opinions on ways to increase public awareness (see Appendix).

A pilot study with seven participants was conducted before the questionnaire was distributed, assessed the questionnaire’s applicability and comprehensibility. The pilot group was excluded from the main study.

Statistical analysis

SPSS Version 24 (IBM Corp., Armonk, NY, USA) was used for data entry and statistical analysis. Descriptive statistics were used to determine the relationships between participants’ demographic data and levels of awareness of patients' rights. p-values <0.05 were considered statistically significant.

## Results

The mean age of the participants was 36 (±13) years. The majority were under 35 years, had completed secondary school education or less, were healthy, had never been admitted to a hospital, undergone hospital follow-ups every six months, and had a single medical record at the MNGHA (Table 1).

**Table 1:** Participants' demographics. SAR, Saudi Arabian Riyals.

Variable	Percentage	Frequency (n)
Gender		
Male	51.6	182
Female	48.4	171
Age		
≤35 years	59.3	207
>35 years	40.7	142
Education		
Illiterate	7	25
School	52.5	188
University	40.5	145
Monthly income		
<5000 SAR	50.3	180
≥5000 SAR	49.7	178
Chronic disease		
No	68.2	244
Yes	31.8	114
Number of admissions		
Never admitted	70.1	244
1-3 times	20.1	70
>3 times	9.8	34
Frequency of hospital visits		
Monthly	30.2	108
Every 6 months	36.3	130
Rarely	33.5	120
Medical records at another hospital		
No	69	247
Yes	31	111

Awareness of a patients’ bill of rights

**Figure 1:**
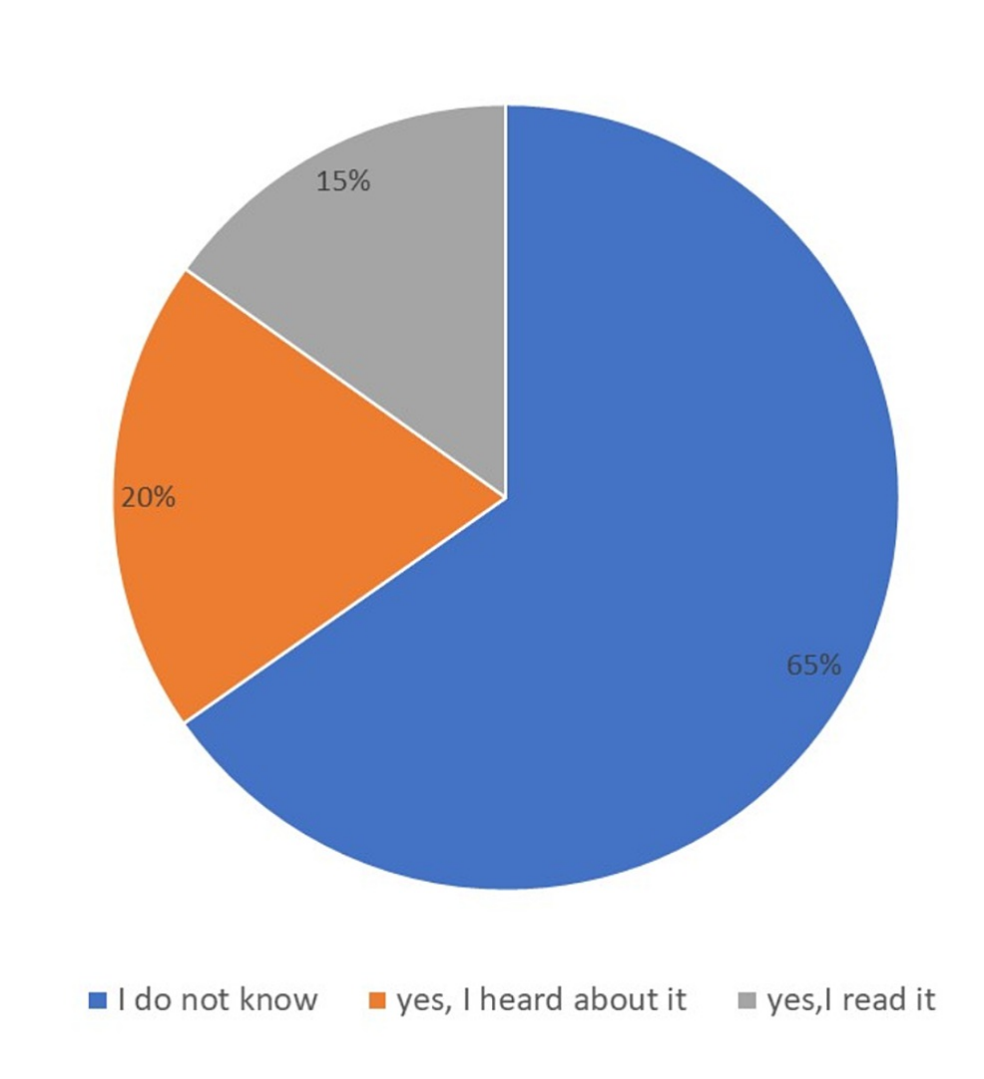
Awareness of patients' bill of rights existence.

Near two-thirds of the participants (65.3%) did not know about the PBR, a fifth (20%) had heard of it and only 15% had actually read the bill (Figure 1).

Source of information

**Figure 2:**
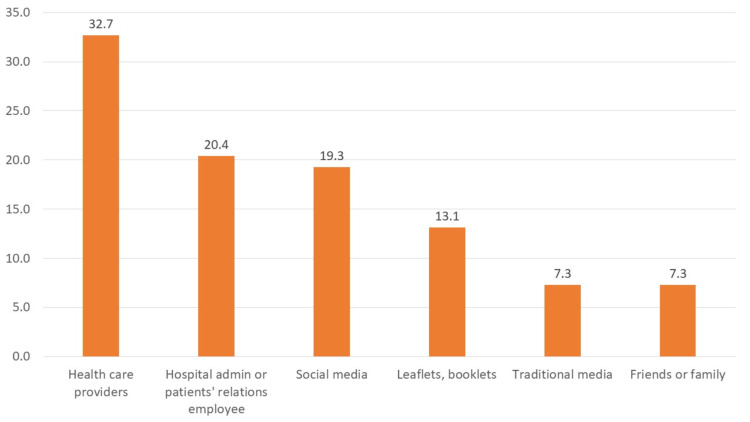
The sources of patients’ rights information.

The most common information source cited by participants (32.7%) was healthcare providers. For 20.4% of participants information came from hospital administration or patient relations departments, and 19.3% of stated that social media or the Internet was their main information source. Traditional mass media, family, friends, and hard-copy materials were the least common sources cited by participants (Figure 2).

Awareness of rights and confidentiality

The overall mean of patients’ awareness was 51.77 ± 8.2 out of 84 and most (72.4%) were moderately aware while 27.5% had excellent awareness. On sub-scale of awareness of medical records confidentiality, the mean score was 13.53 ± 3.88 out of 20 and more than half (52.2%) showed excellent awareness.

Table 2 presents the distribution of responses for each statement of the scale. More than ninety percent agreed that patients should be informed of rights in a comprehensible manner, receive proper healthcare, be respected by healthcare providers, and know healthcare providers’ identities. Between 60% and 90% agreed they had the right to sufficient information about their condition, the right to appropriate assessment and management of pain, the right to submit complaints or suggestions, and the responsibility for their own decisions.

**Table 2:** Participants' awareness of their rights. NGHA: National Guard Health Affairs.

No:	Questions regarding patients' awareness of their rights	Correct answer %(n)	Undecided %(n)	Wrong answer %(n)
1	Healthcare centers should inform patients of their rights in a manner they can understand.	93.6 (335)	1.7 (6)	4.7 (17)
2	My family and I have the right to be provided with the medical services available in NGHA facilities.	98.6 (353)	0.3 (1)	1.1 (4)
3	The doctor should respect the cultural, spiritual, and religious values of the patient.	96.9 (345)	2.5 (9)	0.6 (2)
4	The patient has the right to know the identity and professional status of the healthcare providers (nurses and doctors).	93.6 (335)	2.2 (8)	4.2 (15)
5	In the Emergency Room, Saudi Arabian patients are treated before patients of other nationalities.	20.5 (73)	16.5 (59)	63 (225)
6	The doctor is the only person who can determine the correct investigation and treatment plan, and the patient is not allowed to share their opinion.	16.3 (58)	9.1 (32)	74.6 (265)
7	The physician is not obliged to tell the patient everything about their health status, such as the complications or adverse effects of medications.	70.9 (254)	7.3 (26)	21.8 (78)
8	The physician must assess and manage a patient’s pain, even if he or she has many patients in the clinic.	87.7 (314)	7.8 (28)	4.5 (16)
9	The medical team has the right to provide proper medical or surgical interventions without patient consent.	44.8 (160)	9.5 (34)	45.7 (163)
10	Case: A diabetic patient refuses to take his medications, so the physician gave him diabetes medication and convinced him that it was vitamins. Do you agree with the doctor’s behavior?	21.9 (78)	8.4 (30)	69.7 (248)
11	The patient is responsible for their decision if they refuse medical care advice.	84.6 (303)	5.6 (20)	9.8 (35)
12	Case: A patient is newly diagnosed with a sexually transmitted infection, so the doctor tells his wife in order to prevent transmission. Do you agree with the doctor’s behavior?	3.7 (13)	3.6 (13)	92.7 (332)
13	The hospital has the right to stop taking care of a patient if they refuse treatment.	46.6 (167)	16.8 (60)	36.6 (131)
14	The patient does not have the right to obtain a second opinion.	48.6 (174)	14.5 (52)	36.9 (132)
15	The patient does not have the right to record a verbal or written complaint or suggestion to the healthcare center.	62.6 (224)	9.5 (34)	27.9 (100)
16	The patient should participate in any research studies that are being conducted at the hospital.	18.4 (66)	21.5 (77)	60.1 (215)
B	Which of the following have the right to see your medical record without your permission?			
17	My work manager.	75.6 (270)	5.3 (19)	19 (68)
18	My medical team.	91.9 (329)	2 (7)	6.1 (22)
19	The hospital quality management program team.	49.7 (178)	9.8 (35)	40.5 (145)
20	People with written authorization from the patient or legal authorities.	60.5 (216)	10.6 (38)	28.9 (103)
21	The hospital health facility research team.	55.9 (200)	10.9 (39)	33.2 (119)

Approximately 50% were unaware of their right to informed consent before an intervention, continued medical care after refusing a treatment, and a second opinion. Finally, 60-90% were unaware of their right to receive healthcare without racial discrimination, participate in developing a management plan, refuse treatment, have information kept confidential, and accept or refuse to participate in research studies.

Awareness and demographics associations

University graduates had better awareness compared with participants with lower educational levels (p=0.00). Moreover, there was a significant positive correlation between awareness and monthly income (p=0.003). Awareness and the number of hospital visits participants had made were positively correlated (p=0.015). There were no other significant associations (Table 3).

**Table 3:** Comparison of differences in awareness levels and participants’ demographic data (n=351). $: Names of primary health care centers where data was collected. SAR: Saudi Riyals; PHCC: primary healthcare center.

Variable	Awareness levels		p-value
	Moderate % (n)	Good % (n)	
PHCC			
KashmAlaan^$^	80 (172)	20 (43)	<0.001
Umm Al Hamam^$^	57.5 (46)	42.5 (34)	
IskanYarmouk^$^	64.3 (36)	35.7 (20)	
Gender (n=346)			
Male	69.6 (126)	30.4 (55)	0.25
Female	75.2 (124)	24.8 (41)	
Age (n=342)			
≤35 years	73.4 (149)	26.6 (54)	0.465
>35 years	69.8 (97)	30.2 (42)	
Education			
Illiterate	72 (18)	28 (7)	<0.001
School	81 (149)	19 (35)	
University	61.3 (87)	38.7 (55)	
Income			
<5000 SAR	79.4 (139)	20.6 (36)	0.003
≥5000 SAR	65.3 (115)	34.7 (61)	
Chronic disease			
No	72.5 (174)	27.5 (66)	0.934
Yes	72.1 (80)	27.9 (31)	
Number of admissions (n=350)			
Never admitted	72.0 (180)	28.0 (70)	0.912
1–3 times	74.2 (49)	25.8 (17)	
>3 times	70.6 (24)	29.4 (10)	
Frequency of hospital visits			
Monthly	73.3 (77)	26.7 (28)	0.015
Every 6 months	64.1 (82)	35.9 (46)	
Rarely	80.5 (95)	19.5 (23)	
Medical records at another hospital			
No	73.0 (168)	27.0 (66)	0.711
Yes	71.0 (76)	29.0 (31)	
Awareness of MNGHA PBR existence (n=350)			
I don't know	73 (165)	27 (61)	0.09
Yes, I heard about it	78.6 (55)	21.4 (15)	
Yes, I read about it	61.1 (33)	38.9 (21)	

## Discussion

We found that, despite MNGHA publishing its PBR in 1989, the majority of participants (65.3%) in this study had never heard of it. Similar findings have been reported in previous studies. Almoajel [10] found that most hospitalized patients (74.8%) were ignorant of the MOH’s PBR. Only 21.9% of Alghanim’s [14] study subjects knew the bill existed. Both studies were conducted in Riyadh hospitals. Similar results have been obtained in other Saudi Arabian cities. Al-Mosa and Al-Ghamdi [11] reported that, in Jeddah, 92.3% of patients with non-communicable chronic diseases attending eight PHCCs had not heard about the PBR. Almalki et al [15] concluded that around half of their patients in Taif were not fully aware of the PBR and its contents. Together, these studies indicate poor awareness of the existence of the PBR in the Saudi Arabian population. This is an important finding and might indicate poor dissemination of information regarding such bills with the clients in health care facilities. Al-Mosa and Al-Ghamdi [11] found that only two of eight PHCCs displayed posters on patients’ rights. Similarly, Almoajel [10] found that the largest government hospital exhibited no posters and provided no lessons on the topic. This suggests that the need to inform patients about their rights is ignored by both the healthcare system and healthcare providers calls for proper policy regarding raising awareness of patients’ rights through proper mechanisms.

Surprisingly, although overall awareness of PBR existence was poor, 72.4% of respondents demonstrated a moderate level of awareness of patients’ rights, signifying that people were aware of important aspects of patients’ rights: privacy, proper care, safety, respect, and recognition of social and cultural rights. Consistent with this study, three others found that most patients had a moderate level of awareness regarding patients’ rights [3,10,16]. Habib and Al‐Siber [17] assessed patients’ awareness of 14 items related to patients’ rights, and found that more than one-third were aware of 6-9 rights, and another third were aware of 10-13. This moderate level of awareness among three fourth of participants despite majority did not read the bills might have resulted from high level of education and overall cultural values in Saudi Arabia.

Predictably, we found a positive correlation between awareness scores and having read the PBR: participants who had read the bill tended to score higher than those who had not (p=0.09). Similarly, Almalki et al [15] and Yaghobian et al [9] reported a significant positive association between awareness scores and reading the PBR, demonstrating that the problem is not the structure, length, or content of the bill, but its availability to patients: once patients read the bill, awareness improves.

We found a significant positive association between awareness scores and educational level, income, and regular hospital visits. Other studies have also found similar significant positive associations [5,7-9,11,12,15,17], perhaps because people with higher educational levels are more likely to seek out medical information, explain their wishes to a physician, and complain if their rights are violated [18]. The significant positive association between the number of hospital visits and awareness scores can be explained by high exposure to medical services.

We found that participants were fully aware of some rights but had no knowledge of others. Patient autonomy is a fundamental concept in medical ethics: people agree with the concept in principle, but some disagree with aspects of its application, as evident here. For example, 74.6% of participants believed patients should not be allowed to participate in their management plan, 45.7% agreed that medical teams should provide medical or surgical interventions without patient consent, 69.6% agreed that patients did not have the right to refuse treatment, 36.8% agreed that patients did not have the right to obtain a second opinion, and 60.1% thought participation in research studies was mandatory.

Previous results have highlighted the paternalism of Saudi Arabia’s healthcare system, which led to physicians making decisions without the patient’s consent, or usurping the patient’s role in decision-making. Most patients support this because they believe physicians possess superior knowledge and experience.

The WHO [1] asserts that all people should have equal access to the highest attainable level of healthcare. However, 63% of participants in this study agreed with the statement that Saudi Arabian patients should have priority to health care in Saudi Arabia over patients of other nationalities. This indicates that public education is necessary regarding healthcare equality as an essential human right.

The results regarding participants’ information sources were similar to those in Habib and Al‐Siber [17] and Almoajel [10], which reported that nurses and doctors were the main sources of information. Despite most participants stating they obtained information from healthcare providers, most achieved lower scores than did participants in other studies. This suggests that healthcare providers do not inform patients about their rights, inform them incorrectly, or receive inaccurate information themselves. When comparing awareness scores with sources of information, we found that participants with the highest awareness scores and highest awareness about medical record confidentiality obtained their information from social media, but this trend did not reach statistical significance. This indicates the importance of new media in raising patient awareness of their rights and responsibilities.

Some authors have highlighted barriers to implementing patients’ rights. An angry response from physicians is a main fear preventing patients from requesting good medical care [7]. Vural found that some patients do not know they can request medical services [7]. Almalki et al. [15] found that poor patient education programs, insufficient and overworked medical staff, and poor patient-doctor relationships were the main barriers to the implementation of patients’ rights. If people do not know their rights, they will not seek legal protection.

This study has some limitations. First, it was conducted at only three PHCCs at the National Guard Hospital in Riyadh; therefore, many PHCCs under the umbrella of the MOH and private hospitals inside and outside Riyadh were not included. Additionally, we used convenience sampling to recruit our participants. These may limit the degree to which results may be generalized to all of Saudi Arabia. Second, patient responsibilities were not investigated, and health insurance is not documented in the MNGHA policy, because the government supports the National Guard Hospital. Finally, we did not investigate awareness among healthcare providers, who play an important role in patients’ rights education.

## Conclusions

Our study found that awareness of patients’ rights in the Saudi population is insufficient, The main source of information on patients’ rights cited by participants was healthcare providers. The level of awareness of participants was significantly affected by educational level, income, and attendance at regular hospital follow-up appointments. This calls for extensive effort and education of care providers and patients to achieve satisfactory awareness of these rights. Use of social media is very high in Saudi Arabia and this could provide an effective medium to educate the population at mass level. PBR can be posted in health care facilities for visitors to read. However, further research is required to establish the most effective mechanisms for improving awareness of patients’ rights in Saudi Arabia in general, and among patients and healthcare providers in particular.

## Appendices

Questionnaire

Awareness of patients' Rights Among Saudi Primary Health Care Centers Visitors in Riyadh, Saudi Arabia

Site of data collection: 1- Kashm alaan      2- Umm alhamam     3- Iskan 

Serial number:_______

First part: personnel Data

1.     what is your gender?

a)     male 

b)    female

2.     How old are you? ……... years

3.     what is your level of education?

a)    illiterate

b)    primary school

c)    intermediate school

d)    secondary school 

e)    university 

f)     postgraduate degree

4.     How much your income in Saudi Riyals?

a)     less than 5000 Saudi Riyal

b)    5000 to 15000 Saudi Riyal 

c)     more than 15000 Saudi Riyal

5.     Do you have any chronic disease like (DM - hypertension - hypothyroidism -asthma)?

a)    Yes                        mention it: …………………………….

b)    No     

6.     Have you ever been admitted to hospital (include admission for labors)?

a)     Yes                        How often: …………………………….

b)    No     

7.     How many times you visit hospital or PHC?

a)    Monthly  

b)    every six months

c)    annually 

d)    rarely

8.     Do you have medical record in hospital other than National guard hospital?

a)     Yes

b)    No 

9.     Do you know about patient's bill of rights that published by Ministry of National Guard?

a)     Yes, I read it

b)    Yes, I heard about it 

c)      I don't know anything about it.

Second part:  Read the statement below carefully, and put tick mark to know how much your awareness of patient right.

Questions regarding patients' awareness of their rights. Please indicate on scale 0-5 . Where “0” means strongly disagree and “5” means strongly agree.

10.           Healthcare centers should inform patients of their rights in a manner they can understand.

11.           My family and I have the right to be provided with the medical services available in NGHA facilities.

12.           The doctor should respect the cultural, spiritual, and religious values of the patient.

13.           The patient has the right to know the identity and professional status of the healthcare providers (nurses and doctors).

14.           In the Emergency Room, Saudi-Arabian patients are treated before patients of other nationalities.

15.           The doctor is the only person who can determine the correct investigation and treatment plan, and the patient is not allowed to share their opinion.

16.           The physician is not obliged to tell the patient everything about their health status, such as the complications or adverse effects of medications.

17.           The physician must assess and manage a patient’s pain, even if he or she has many patients in the clinic.

18.           The medical team has the right to provide proper medical or surgical interventions without patient consent.

19.         Case: A diabetic patient refuses to take his medications, so the physician gave him diabetes medication and convinced him that it was vitamins. Do you agree with the doctor’s behavior?

20.         The patient is responsible for their decision if they refuse medical care advice.

21.         Case: A patient is newly diagnosed with a sexually transmitted infection, so the doctor tells his wife in order to prevent transmission. Do you agree with the doctor’s behavior?

22.         The hospital has the right to stop taking care of a patient if they refuse treatment.

23.         The patient doesn't have the right to obtain a second opinion.

24.         The patient doesn't have the right to record a verbal or written complaint or suggestion to the healthcare center.

25.         The patient should participate in any research studies that are being conducted at the hospital.

Who of the following have the right to see your medical record without your permission?

26.         My work manager.

27.         My medical team.

28.         The hospital quality management program team.

29.         People with written authorization from the patient or legal authorities.

30.         The hospital health facility research team.

Third part: Read the statement below carefully, and put tick mark on the statement of your choice.

31          Choose one: What is your main source of patients’ rights information        Response

A.                         Old Media (TV - newspaper- radio)        

B.                         Doctor, nurse or other health care provider        

C.                         New media e.g., social media in Internet.            

D.                         Booklet, brochure or poster boards.       

E.                         Family or friends.           

F.                          Hospital administration or patients’ relations.    

32          Choose one: Which of following statements consider highly effective in increasing awareness of patients’ rights among population in your opinion?

A.                         Increase leaflet and booklet in waiting area.

B.                         Make videos or websites and spread them in internet and TV.

C.                         Special Office in every hospital for patients’ rights educations.

D.                         include this topic in school and university curriculum.

E.                         Educate and evaluate the healthcare providers to respect patients’ rights.
